# Critical Involvement of the Thioredoxin Reductase Gene (*trxB*) in *Salmonella* Gallinarum-Induced Systemic Infection in Chickens

**DOI:** 10.3390/microorganisms12061180

**Published:** 2024-06-11

**Authors:** Zhihao Zhu, Zuo Hu, Shinjiro Ojima, Xiaoying Yu, Makoto Sugiyama, Hisaya K. Ono, Dong-Liang Hu

**Affiliations:** 1 Department of Zoonoses, Kitasato University School of Veterinary Medicine, Towada 034-8628, Japan; zhuzhihao@webmail.hzau.edu.cn (Z.Z.); pegplus12138jp@gmail.com (Z.H.); s-ojima@niid.go.jp (S.O.); yuxiaoying1992@swu.edu.cn (X.Y.); hisaono@vmas.kitasato-u.ac.jp (H.K.O.); 2 College of Veterinary Medicine, Huazhong Agricultural University, Wuhan 430070, China; 3 Research Center for Drug and Vaccine Development, National Institute of Infectious Diseases, Tokyo 162-8640, Japan; 4 College of Veterinary Medicine, Southwest University, Chongqing 400715, China; 5 Laboratory of Veterinary Anatomy, Kitasato University School of Veterinary Medicine, Towada 034-8628, Japan; masugi@vmas.kitasato-u.ac.jp

**Keywords:** *Salmonella* Gallinarum, thioredoxin reductase, pathogenicity, chicken

## Abstract

*Salmonella enterica* serovar Gallinarum biovar Gallinarum (SG) causes fowl typhoid, a notifiable infectious disease in poultry. However, the pathogenic mechanism of SG-induced systemic infection in chickens remains unclear. Thioredoxin reductase (TrxB) is a redox protein crucial for regulating various enzyme activities in *Salmonella* serovar, but the role in SG-induced chicken systemic infection has yet to be determined. Here, we constructed a mutant SG strain lacking the *trxB* gene (*trxB*::Cm) and used chicken embryo inoculation and chicken oral infection to investigate the role of *trxB* gene in the pathogenicity of SG. Our results showed that *trxB*::Cm exhibited no apparent differences in colony morphology and growth conditions but exhibited reduced tolerance to H_2_O_2_ and increased resistance to bile acids. In the chicken embryo inoculation model, there was no significant difference in the pathogenicity of *trxB*::Cm and wild-type (WT) strains. In the chicken oral infection, the WT-infected group exhibited typical clinical symptoms of fowl typhoid, with complete mortality between days 6 and 9 post infection. In contrast, the *trxB*::Cm group showed a 100% survival rate, with no apparent clinical symptoms or pathological changes observed. The viable bacterial counts in the liver and spleen of the *trxB*::Cm-infected group were significantly reduced, accompanied by decreased expression of cytokines and chemokines (IL-1β, IL-6, IL-12, CXCLi1, TNF-α, and IFN-γ), which were significantly lower than those in the WT group. These results show that the pathogenicity of the *trxB*-deficient strain was significantly attenuated, indicating that the *trxB* gene is a crucial virulence factor in SG-induced systemic infection in chickens, suggesting that *trxB* may become a potentially effective target for controlling and preventing SG infection in chickens.

## 1. Introduction

*Salmonella enterica* serovar Gallinarum biovar Gallinarum (SG) is a Gram-negative facultative anaerobic rod bacterium belonging to the family *Enterobacteriaceae*. It is the causative agent of fowl typhoid, a severe septicemic and fatal systemic infection in poultry [1,2]. Fowl typhoid poses significant economic losses to the poultry industry due to its high mortality rate, and it has been designated as a notifiable infectious disease under the Livestock Infectious Diseases Control Law, referred to as SG infection [3]. In recent years, this infectious disease has become widespread in developing countries in Central and South America as well as in East Asia, which are major poultry production and export countries [4]. Although vaccination has been attempted to induce immunity against SG for fowl typhoid control—for example, attenuated strains of SG have been used as live vaccines—the pathogenicity of these vaccine strains is not entirely lost, leading to inadequate preventive efficacy [5,6,7]. Further understanding of the pathogenesis and infection mechanisms of SG is essential for the development of safer and more effective vaccines [8,9]. Enhanced understanding of SG’s virulence factors, host–pathogen interactions, and immune evasion strategies will facilitate the design of novel vaccine candidates capable of eliciting robust and lasting protective immunity against fowl typhoid. This pursuit not only holds promise for mitigating economic losses within the poultry industry but also contributes to safeguarding public health and food security on a global scale.

Thioredoxin (Trx) is a redox protein that controls various enzyme activities within bacteria and responds to oxidative stress by reducing and cleaving disulfide bonds in other proteins [10]. The active site of thioredoxin contains a -Cys-X-X-Cys- sequence with two forms: an oxidized form with a disulfide bond between two Cys residues and a reduced form with free thiol groups. Thioredoxin reductase, together with thioredoxin and NADPH, forms the thioredoxin system. Its function is to reduce intracellular proteins serving as substrates while oxidizing itself, thereby regulating cellular functions that may play an important role in bacterial pathogenesis. Previous studies have demonstrated that many bacterial virulence factors require stable disulfide bonds for proper folding and function [11,12,13]. In *Escherichia coli* (*E. coli*), Trx plays a crucial role in oxidative protein folding by donating electrons to the disulfide bond system and contributing to the isomerization of incorrectly paired disulfide bonds [14]. The Trx domain-containing oxidase is responsible for introducing disulfide bonds into substrate proteins and subsequently obtaining the oxidizing equivalent from membrane proteins [15]. The disulfide bond formation system in *E. coli* catalyzes the oxidative folding step and consists of several enzymes that form two distinct pathways: oxidative and isomerization [16]. The *trxB* gene, encoding thioredoxin reductase, is required for *Neisseria gonorrhoeae* to defend against oxidative and disulfide bond stress [11,12]. Several studies have reported that *Listeria monocytogenes* thioredoxin A (TrxA) contributes to bacterial virulence and motility through redox interactions [13], and the pathogenicity of *Salmonella* Typhimurium with mutant *trxB* gene is significantly reduced [11,17]. These studies demonstrate the importance of detailed studies of oxidoreductases to understand the underlying mechanisms of bacterial pathogenesis and the potential for the development of novel therapeutics. To date, however, components of the Trx system of *Salmonella* Gallinarum, a chicken-specific pathogen, have not been characterized, and its role in SG-induced systemic infection in chicken remains unclear.

In this study, we employed mutant *S.* Gallinarum 287/91 strain lacking the *trxB* gene (*trxB*::Cm) and utilized chicken embryo inoculation and chicken oral infection models to investigate whether the *trxB* gene is involved in the SG-induced chicken systemic infections and determine the role of the *trxB* gene in modulating the immune response during SG-induced infection.

## 2. Materials and Methods

### 2.1. Bacterial Strains, Plasmids, and Growth Conditions

The wild-type strain used in this study was *S.* Gallinarum 287/91 (hereafter referred to as WT); the complete genome has been previously published [18]. The WT strain was maintained at −80 °C in Luria–Bertani (LB) broth supplemented with 30% glycerol and revitalized in LB broth (Eiken Chemical, Tokyo, Japan) with shaking at 150 rpm at 37 °C. For infection experiments, both the WT strain and deletion mutant strain were cultured in LB broth at 37 °C to the logarithmic phase, then harvested by centrifugation, and washed twice with sterile 0.01 M phosphate-buffered saline (PBS).

### 2.2. Construction of trxB::Cm Mutant

The mutant strain construction was conducted using the λ-red recombination method [19], employing plasmids pKD46 (GenBank: MF287367.1) and pKD3 (GenBank: AY048742.1). The pKD46-harboring WT strain was constructed using the electroporation method and served as the recipient. The primers (F1 and R1) designed to pKD3 are listed in Table 1. The PCR was performed in a T100TM Thermal cycler (Bio-Rad, Hercules, CA, USA) with an initial denaturation at 94 °C for 2 min, followed by 30 cycles of 98 °C for 10 s, 55 °C for 30 s, and 68 °C for 1 min. The PCR product was introduced into the recipient strain by electroporation, then spread on Luria–Bertani (LB) agar (Eiken Chemical, Tokyo, Japan) plates containing 20 µg/mL chloramphenicol, and incubated at 37 °C. The colonies obtained were further streaked on LB agar plates without chloramphenicol and incubated overnight at 42 °C to eliminate the pKD46. After identification of the deletion of the *trxB* gene with primers (F2 and R2) listed in Table 1, the resulting strain was named as the *trxB*::Cm strain.

### 2.3. Assays of Growth Kinetics and Sensitivity

To compare the growth changes of the WT strain and the trxB deletion mutant strain under oxidative stress and intestinal environments as well as the changes in the resistant to nalidixic acid, we measured the growth kinetics and sensitivity of the two strains in LB broth supplemented with hydrogen peroxide, bile acids, or nalidixic acid. Under the same bacterial density, 100 µL of the bacterial liquid (OD_600_ = 1.5) was inoculated into 10 mL of LB broth and cultured at 37 °C to measure the absorbance (OD_600_ nm) at 2, 4, 6, 8, 10, 12, and 24 h post inoculation. Meanwhile, 100 µL of the bacterial liquid (OD_600_ = 1.5) was spread on LB agar plates to count the colonies at 2, 4, 8, 10, and 24 h post inoculation. The colony morphology and bacterial shape were observed on LB agar plates and under a microscope (OLYMPUS BX41, Olympus Corporation, Tokyo, Japan) after culturing on LB agar plates for 12 h at 37 °C. Bacterial growth in LB broth containing bile acids (0.01, 0.02, 0.04, and 0.08 mM), nalidixic acid (1.25, 2.5, and 3.25 μg/mL), or hydrogen peroxide (0.5, 1, and 2 mM) was observed using the above methods at 12 h. The percentage ratio (×100) of absorbance measurements after cultivation in the reagent-added LB to those after cultivation in LB alone was calculated, and growth under different conditions was compared between the strains.

### 2.4. Chicken Embryo Infection Model

SPF chicken embryos were incubated and developed in an incubator at 37 °C for 10 days with turning every hour. Subsequently, they were divided into the NC group (n = 5), the WT group (n = 5), and the *trxB*::Cm group (n = 5) and inoculated with the bacteria strains adjusted to 1 × 10^3^ CFU/egg (PBS for the NC group). The survival of chicken embryos was observed using candling lights every day. Additionally, WT-inoculated and *trxB*::Cm-inoculated chicken embryos underwent sampling of allantoic fluid and embryo on days 1 and 3 post bacterial inoculation. Allantoic fluid (1 mL) was collected in a 1.5 mL centrifuge tube with 10-fold serial dilutions to spread on LB agar plates at 37 °C for counting the next day. The embryos were each collected in a 1.5 mL centrifuge tube, and their weights were measured using an electronic balance. The recorded weights served as references for adding nine times the volume of 1 × PBS to the tube. Homogenization was achieved using a pestle, and the resulting homogenate was serially diluted 10-fold to spread on LB agar plates at 37 °C for counting the next day.

### 2.5. Chicken Oral Infection Model

We utilized Boris brown hens (20 days old, Aomori Poultry Co., Ltd., Aomori, Japan) as the experimental chickens. The chicken experiments were conducted after approval by the Kitasato University Animal Experiment Committee and obtaining the authorization from the university president (Approval Number: 20-055 and 21-039). WT and *trxB*::Cm strains were individually adjusted to 1 × 10^8^ CFU/mL in PBS. One milliliter of the bacteria suspension was orally administered to each chicken using a sterile pipette. The non-infected control group received 1 mL of PBS via oral administration. Chickens (n = 3 to 6 for each group) infected with each bacterial strain were observed and recorded daily until day 14 post inoculation to compare their survival rates. As described in the method in our previous study [20], liver and spleen samples were collected from both WT- and *trxB*::Cm-inoculated chickens on days 1, 3, 5, and 7 post inoculation, followed by fixation, preparation of paraffin sections, hematoxylin and eosin (HE) staining, and observation using a light microscope (Olympus BX41, Olympus Co., Ltd., Tokyo, Japan), and the magnification was 100× or 400×. These infection experiments were performed at the same time using the WT and *trxB*::Cm strains as well as two other mutant strains (*wecB*::Cm and mSPI-14) that are not shown in this article.

### 2.6. Quantitative Real-Time PCR Analysis

To analyze the host responses in the organs of the chickens infected with the WT or *trxB:*:Cm mutant, four or five chickens in each group were euthanized at 3 and 5 days post infection. Tissue samples of the liver were immersed separately in 0.5 ml of RNA later (Thermo Fisher Scientific, Waltham, MA, USA) and stored at −80 °C until use. Total RNA was extracted from 5 × 5 mm of the tissue using RNAiso Plus (TaKaRa, Kusatsu, Japan) according to the manufacturer’s instructions. The quantity and quality of RNA were determined by spectral analysis (NanoDrop 2000, Thermo Fisher Scientific, WI, USA). After being treated with DNase, RNA was transcribed to complementary DNA (cDNA) using the ReverTra Ace R qPCR RT Master Mix (Toyobo, Tokyo, Japan), following the manufacturer’s instructions [21]. The expression of mRNA for interleukin (IL)-1β, IL-6, tumor necrosis factor (TNF)-α, interferon (IFN)-γ, IL-12, and CXCLi1 in the tissues was measured using quantitative real-time RT-PCR, using the primers shown in Table 2. The expression of the genes was determined using the threshold cycle (ct) value relative to that of the housekeeping gene GAPDH, and the results were expressed as fold-changes in corrected target gene in the infected chickens relative to the uninfected controls.

### 2.7. Statistical Analysis

Bacterial counts in organs were logarithmically transformed, and the significant differences in mean values between each day post inoculation were assessed using one-way analysis of variance (ANOVA) with Tukey’s multiple comparison test. For the analysis of cytokine expression levels, Dunnett’s test was employed to examine the significant differences between the expression levels at each day post inoculation and the expression level in the non-infection control. All statistical analyses were performed using GraphPad Prism version 9.2.0 (GraphPad Software, San Diego, CA, USA), and significance was determined when the *p*-value was below 0.05.

## 3. Results

### 3.1. Deletion of trxB Gene Does Not Affect the Morphology and Growth

**Figure 1:**
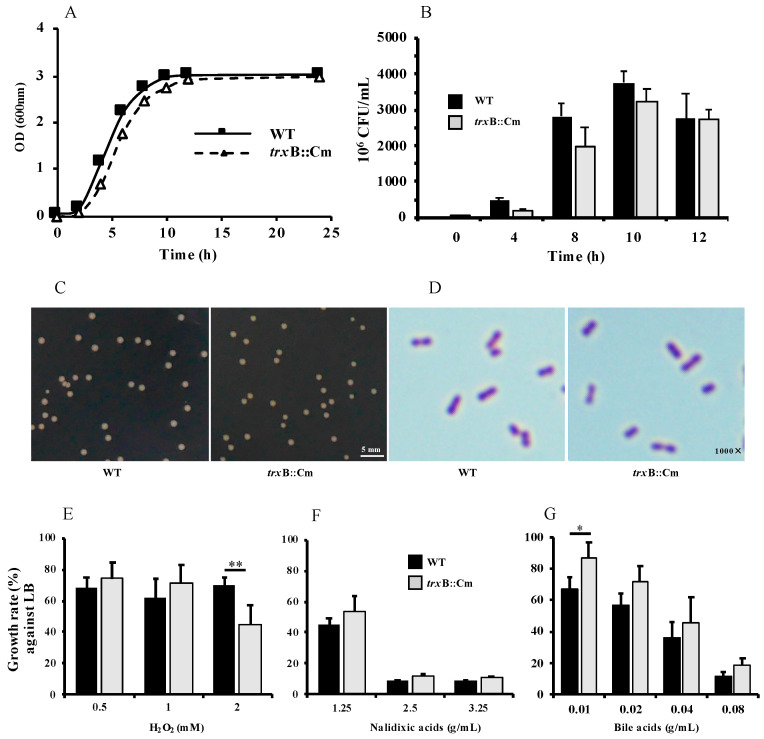
Comparison of characteristics between wild-type (WT) and mutant *trxB*::Cm strains. (**A**) Growth curves based on OD_600_ and (**B**) bacterial counts. (**C**) Colonies on LB agar plates and (**D**) Gram-stained images (1000×, Olympus, BX41, Olympus Co., Ltd., Tokyo, Japan) of WT and trxB::Cm grown on LB agar plates. Growth rates (%) of WT and *trxB*::Cm in LB medium supplemented with (**E**) H_2_O_2_, (**F**) nalidixic acid, and (**G**) bile acids after 12 h of incubation, normalized to growth in LB medium. * *p* < 0.05; ** *p* < 0.01.

To study the role of the *trxB* gene in SG, we constructed a *trxB*::Cm deletion mutant strain by the λ-red recombination method. There were no significant differences in growth between the WT and *trxB*::Cm strains as measured in LB broth by bacterial density (Figure 1A) and on LB ager plates by bacterial counts (Figure 1B). And there were no differences in colony size and morphology between the WT and *trxB*::Cm strains after 12 h cultivation (Figure 1C,D). In LB broth supplemented with 2 mM H_2_O_2_, the growth of the *trxB*::Cm strain was significantly reduced compared to the WT strain (*p* < 0.01, Figure 1E). In LB broth supplemented with nalidixic acid, there was no significant difference between the two strains at any concentration (Figure 1F). However, in the bile acid-supplemented LB broth, the *trxB*::Cm strain exhibited a significant increase in proliferation at 0.01 mM compared to the WT strain at 12 h post culture (*p* < 0.01, Figure 1G).

### 3.2. Deletion of trxB Gene Did Not Affect the Pathogenicity of SG in Chicken Embryo Infection

**Figure 2:**
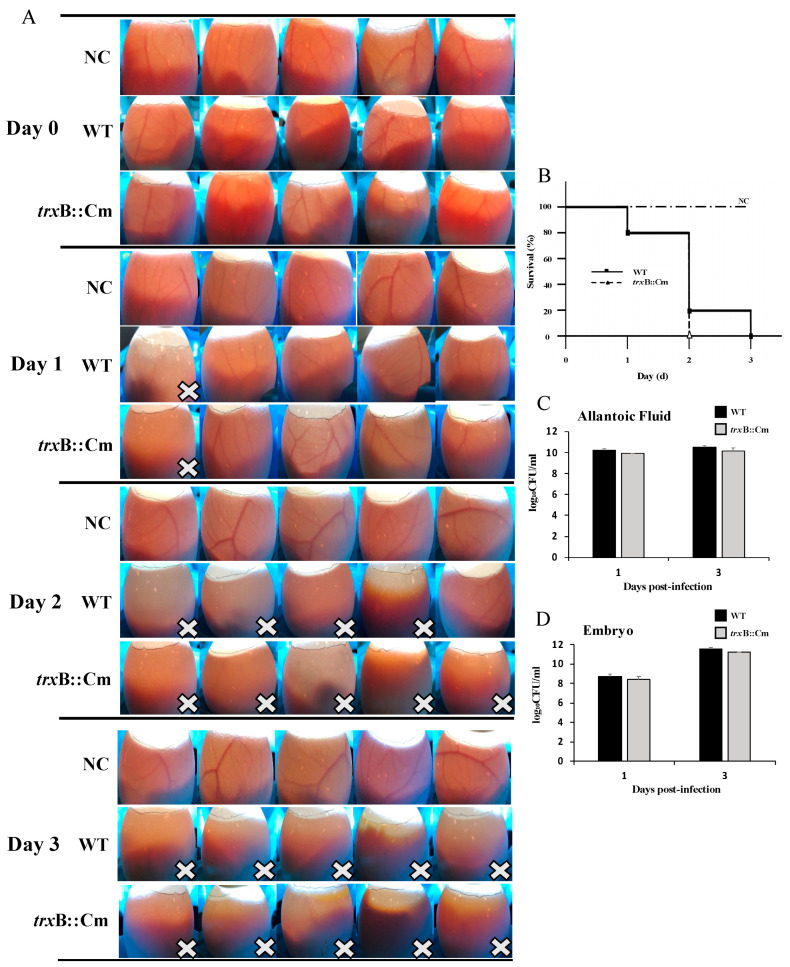
Comparison of pathogenicity in chicken embryo inoculation model between negative control (NC) group, wild-type (WT) group, and the mutant *trxB*::Cm group. (**A**) Survival status of chicken embryos after inoculation with PBS, WT, and *trxB*::Cm. The “×” means the death of the embryos. (**B**) Survival curves of chick embryos inoculated with WT or *trxB*::Cm. And bacterial counts in the allantoic fluid (**C**) and embryos (**D**) of chick embryos after inoculation with WT or *trxB*::Cm.

To investigate the pathogenicity of *trxB*::Cm and WT to chicken embryos, the embryos were inoculated with the bacterial suspensions adjusted to 1 × 10^3^ CFU/egg, and the development or death of the embryos was observed for 4 days. The results showed that the WT and *trxB*::Cm groups showed visible blood vessels on day 0, and all embryos survived. Subsequently, a gradual decrease in survival was observed, with all embryos dying on the third day after inoculation (Figure 2A). There were no significant differences between the two groups (Figure 2B).

On days 1 and 3 after inoculation, amniotic fluid and embryos were collected, and bacterial counts were measured. The results also showed no significant difference in bacterial growth between *trxB*::Cm and WT in amniotic fluid and embryos (Figure 2C,D).

### 3.3. Deletion of trxB Gene Significantly Reduced Bacterial Load and Clinical Symptoms in the Chicken Infection Model

**Figure 3:**
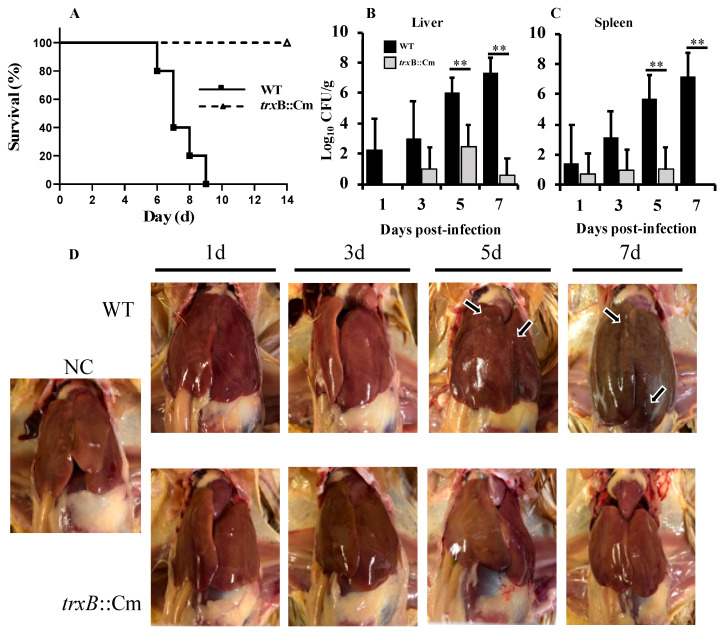
Comparison of pathogenicity in chicken oral infection model between wild-type (WT) group and the mutant *trxB*::Cm group. (**A**) Survival rates of chickens infected with WT or *trxB*::Cm. (**B**,**C**) Bacterial counts in the liver and spleen of each strain-infected chicken: WT-inoculated chickens: 1 day post inoculation (n = 3) and 3, 5, and 7 days post inoculation (n = 6). *trxB*::Cm-inoculated chickens: 1 day post inoculation (n = 3); 3 days post inoculation (n = 6); 5 and 7 days post-inoculation (n = 5). (**D**) Pathological observation of the liver at 1, 3, 5, and 7 days post inoculation, and arrows indicate white pinpoint lesions and small necrotic foci. Graphs represent the mean ± standard deviation of bacterial counts in organs at each post-infection time point. ** *p* < 0.01.

To investigate the pathogenic roles of *trxB* gene in the systemic infection of chickens induced by SG in vivo, we analyzed the mortality and clinical changes in chickens orally infected with the *trxB*::Cm and WT strains. WT-infected chickens exhibited clinical symptoms from day 4 post inoculation, with complete mortality observed between days 6 and 9. In contrast, *trxB*::Cm-infected chickens showed no significant clinical symptoms throughout the 14-day observation period, with all chickens surviving (Figure 3A). In the liver of WT-inoculated chickens, the bacterial count increased continuously from day 1 to day 7, reaching 10⁷ CFU/g. Conversely, the bacterial count in the liver of *trxB*::Cm-infected chickens remained below 10 CFU/g on day 3, 10^2^ CFU/g on day 5, and 10 CFU/g on day 7, significantly lower than the WT group (*p* < 0.01, Figure 3B). Similarly, the bacterial count in the spleen of *trxB*::Cm-infected chickens was significantly lower than that in the WT group, with 10 CFU/g on day 5 and no detectable bacteria on day 7 (*p* < 0.01, Figure 3C).

Macroscopic observations of WT-infected chicken livers revealed significant pathological changes, such as white pinpoint lesions and small necrotic foci on day 5 and liver hypertrophy and congestion causing deepening of coloration on day 7 (Figure 3D). Conversely, *trxB*::Cm-infected chicken livers showed no significant macroscopic changes.

**Figure 4:**
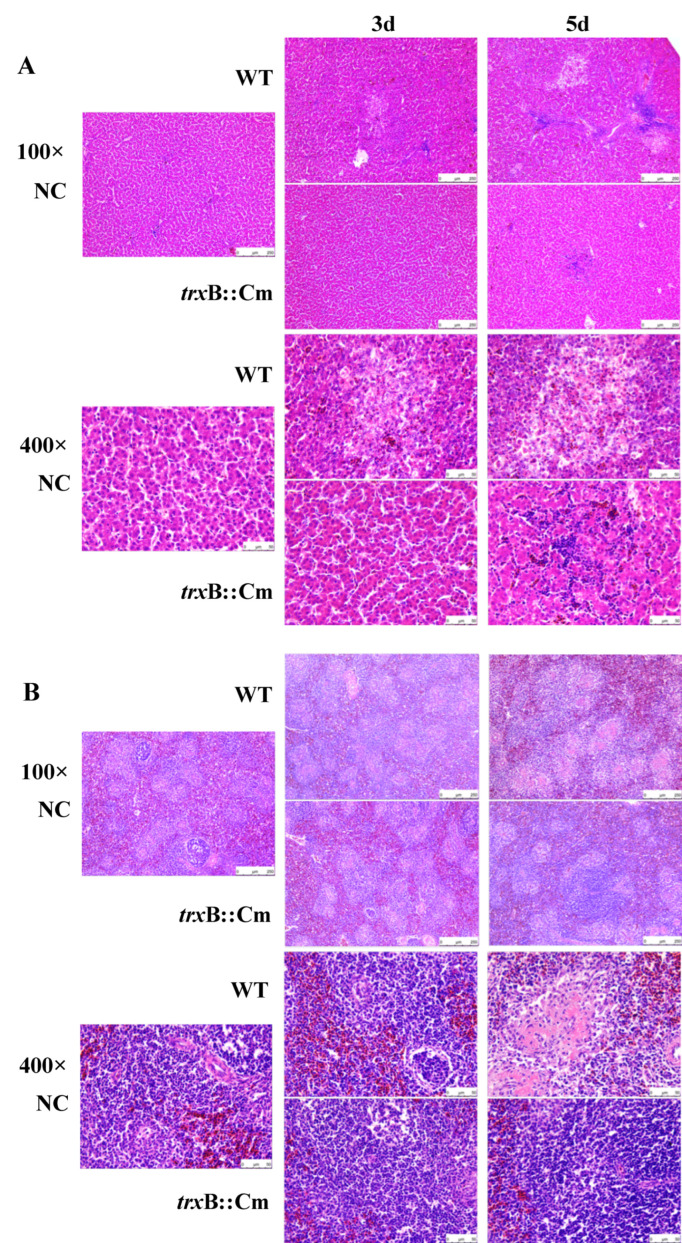
Histopathological images of the liver and spleen of chickens orally infected with wild-type (WT) group and the mutant *trxB*::Cm group. Liver and spleen samples were collected from chickens at day 3 and 5 post infection, and histopathological specimens were prepared from paraffin sections stained with hematoxylin and eosin (HE). Histopathological images of the liver (**A**) and spleen (**B**) under magnification 100× and 400×. NC, non-infected control.

We next performed histopathological examination of livers and spleens of infected chickens to determine whether the *trxB* gene is involved in tissue inflammation caused by SG. Microscopic analysis of liver tissue from WT-infected chickens showed lymphocytic and heterophilic infiltration starting on day 3 and progressing to extensive necrosis by day 5. In contrast, chickens infected with *trxB*::Cm showed mild lymphocytic and heterophilic infiltration with minimal foci of necrosis on day 5 (Figure 4A). In the spleen tissue of WT-infected chickens, multiple coagulative necrosis was observed on day 5. In contrast, the spleen of *trxB:*:Cm-inoculated chickens showed no obvious pathological changes (Figure 4B).

### 3.4. Deletion of trxB Gene Significantly Reduced Lower Level of Immune Response in Chicken Infection Model

**Figure 5:**
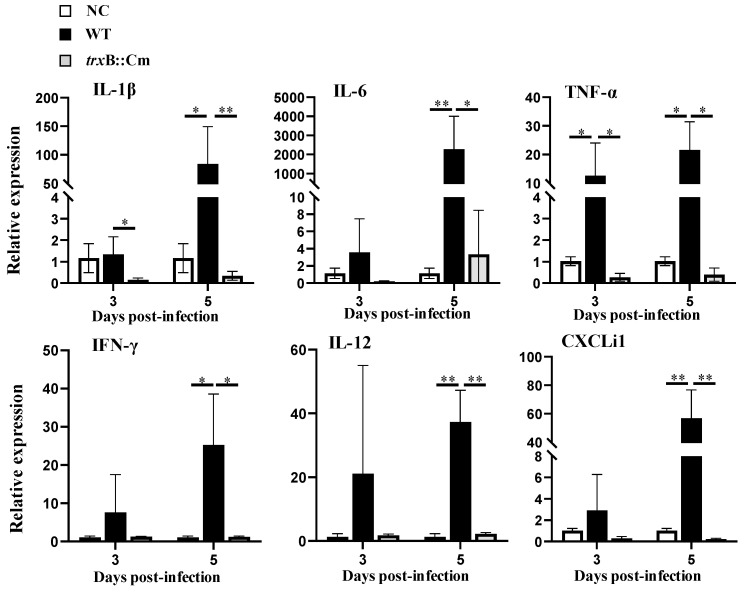
mRNA expression levels of cytokines and chemokines in the liver of chickens infected with wild-type (WT) and the mutant *trxB*::Cm strains (n = 6). mRNA expression levels of cytokines and chemokines in the liver at 3 and 5 days post infection were measured by real-time PCR. Graphs represent the mean ± standard deviation of mRNA expression levels at each time point. Statistical analysis of significant differences between each infection group at each time point was conducted using Tukey’s multiple comparison test. * *p* < 0.05; ** *p* < 0.01.

We further analyzed and compared the immune responses of chickens infected with WT and *trxB*::Cm (Figure 5). In the liver of the WT-infected group, the expression of TNF-α increased significantly at days 3 and 5 post infection compared to the non-infected control group (*p* < 0.05). At day 5 post inoculation, there was also a notable increase in the expression levels of IL-1β, IL-6, IFN-γ, IL-12, and CXCLi1 compared to the non-infected control group. In contrast, the liver of the *trxB*::Cm-infected group showed no significant changes in the expression of any cytokine and chemokine at days 3 and 5 post infection compared to the non-infected control group. Furthermore, the expression levels of IL-1β and TNF-α in the liver of *trxB*::Cm-infected group at day 3 post infection exhibited significantly lower expression compared to the WT-infected group (*p* < 0.05), and at day 5, the expression levels of IL-1β, IL-12, and CXCLi1 (*p* < 0.01) and IL-6, TNF-α, and IFN-γ (*p* < 0.05) were significantly lower than that of the WT-infected group.

## 4. Discussion

In this study, we investigated the relationship of thioredoxin reductase gene (*trxB*) and the pathogenicity of SG in chicken systemic infection, using both a WT and a mutant strain lacking the *trxB* gene (*trxB*::Cm). Our results showed that there were no significant differences in the growth curve and morphology between the *trxB*::Cm and WT, but the *trxB*::Cm showed significantly lower resistance to H_2_O_2_ compared to WT (Figure 1). Although no significant difference in pathogenicity between *trxB*::Cm and WT was observed in the chick embryo infection model, in the chicken oral infection model, the WT group exhibited typical clinical symptoms of fowl typhoid with high mortality, whereas the *trxB*::Cm group showed a 100% survival rate, with no obvious pathological changes in macroscopic observations and mild lymphocytic and heterophilic infiltration with minimal foci of necrosis in histopathological examination. In addition, the viable bacterial counts in the liver and spleen of the *trxB*::Cm group were significantly reduced, and the expression of inflammatory cytokines and chemokines were significantly lower than those in the WT group. These results demonstrated for the first time that the thioredoxin reductase gene (*trxB*) plays a critical involvement in SG-induced systemic infection in chickens.

Thioredoxin is a thioredoxin-dependent peroxidase that exhibits enzyme activity in the presence of reduced thioredoxin, reducing H_2_O_2_ to water [22,23]. Previous studies have demonstrated that in the absence of thioredoxin reductase, reduced thioredoxin cannot be efficiently recycled, leading to decreased resistance to H_2_O_2_ [24,25]. Thioredoxin reductase utilizes the reducing ability of NADPH and indirectly participates in intracellular redox reactions [26,27]. Experimental reports using *E. coli* mutant strains showed an interesting change when only the *trxB* gene was deleted: Thioredoxin acted as an oxidant rather than being re-reduced, resulting in the accumulation of alkaline phosphatase [28]. It has been reported that co-expression of the gene *trxA*, which also participates in protein redox reactions, enhances the expression of epidermal growth factor in a forced protein expression system in *E. coli*, while thioredoxin reductase is responsible for regulating protein expression [29]. Several studies on the thioredoxin system in *E. coli* have shown that oxidative stress can promote the activation and expression of the chaperone protein Hsp33 in *E. coli*, and the *trxB* mutant, lacking components of the thioredoxin system, enhances the resistance to oxidative stress [30,31]. The hyper resistance of an *E. coli trxB* strain to H_2_O_2_ is attributed to constitutive up-regulation of *katG* (catalase/hydroperoxidase I), an element of the OxyR regulon [25]. In the present study, our results also showed that the *trxB*::Cm mutant of SG has reduced resistance to H_2_O_2_ compared with WT and that the reduced resistance of *trxB*::Cm can be attributed to reduced thioredoxin levels, suggesting that SG may have similar defense mechanism against oxidative stress to that of *E. coli*.

In the chicken embryo inoculation model, there was no significant difference in the pathogenicity of *trxB*::Cm and WT. This may be due to the infection route to the developing chicken embryo and the immature development of the chicken’s immune defense system. This is because cellular immunity, which is critical to the immune response of chickens, needs to start working around 20 days after hatching, while humoral immunity needs to start working around 3 to 4 days after hatching [32,33,34]. In this study, we used WT and *trxB*::Cm to inoculate 10-day-old chicken embryos, a period during which the immune system (particularly cellular immunity) is not fully mature and functional during embryonic development. This result shows that the pathogenic role played by *trxB* is closely related to the immune system and organs and tissues in chickens. In the chicken oral infection model, the survival rate, bacterial counts in organs, pathological changes, and cytokine and chemokine expression levels were determined. Our results suggest the reduced pathogenicity of *trxB*::Cm is attributed to its decreased colonization ability in the liver and spleen and lower induction of inflammatory responses. Unlike *S.* Enteritidis and *S*. Typhimurium, *S*. Gallinarum induces a severe, septicemic, and systemic infectious disease in poultry rather than gastrointestinal infections [7,20]. In the systemic infection model in chickens, a key component of SG pathogenesis is the bacterium’s ability to disseminate and colonize various systemic sites. During infection, the bacteria need to efficiently traverse the intestinal barrier, colonize intracellularly, and subsequently spread to surrounding tissues. Cheng et al. also indicated that thioredoxin is essential for motility and contributes to host infection of *Listeria monocytogenes* via redox interactions [13]. The function of *trxB* remains a topic of exploration [35,36]. As a crucial component of the thioredoxin system, *trxB* plays a pivotal role in oxidative stress, virulence modulation, growth regulation, drug resistance, and other essential processes [37,38,39]. The multifunctional nature of *trxB* underscores its importance in microbial physiology and pathogenesis, highlighting its potential as a target for therapeutic intervention and the development of novel antimicrobial strategies.

In conclusion, our findings demonstrate the critical involvement of the *trxB* gene in SG-induced systemic infection in chickens and underscore the potential of *trxB* as a promising target for controlling and preventing SG infection in poultry. Given its crucial role in SG pathogenicity, targeting *trxB* may offer a novel strategy for developing therapeutics or vaccines against fowl typhoid. The attenuated strain lacking *trxB* serves as a vaccine candidate, and assessing its immunogenicity, safety, and efficacy in challenge experiments is the focus of our subsequent work. However, additional research is needed to elucidate the specific mechanisms underlying *trxB*-mediated virulence and to evaluate the feasibility and efficacy of targeting *trxB* in controlling SG infection in poultry populations. Overall, our study contributes to a better understanding of SG pathogenesis and provides valuable insights into the development of strategies to combat fowl typhoid.

## Figures and Tables

**Table 1:** Primers and sequences for the λ-red recombination method.

Primers	Sequences	Amplicon (bp)
SG-F1	ACAATTCTGCTCATTGTCTGCCAACAACTATGGGGATCTCGTGTAGGCTGGAGCTGCTTCTT	1014
SG-R1	TCTATAGTCGCCTTTTTTACTTTTGTTACTGATTTGTAAAAACATATGAATATCCTCCTTAG	
SG-F2	CTCACATCACTGTTCAGAGTCGCTG	764
SG-R2	TTTTCACCATGGGCAAATAT	

**Table 2:** Primers and sequences for RT-qPCR.

Primers	Sequences	Amplicon (bp)	Accession No.
GAPDH-F	GGCACTGTCAAGGCTGAGAA	99	NM_204305.2
GAPDH-R	TGCATCTGCCCATTTGATGT		
IL-1β-F	CGAGGAGCAGGGACTTTGC	71	NM_204524.2
IL-1β-R	GAAGGTGACGGGCTCAAAAA		
IL-6-F	CCTGGCGGCCACGAT	61	NM_204628.2
IL-6-R	CGAGTCTGGGATGACCACTTC		
TNF-α-F	GAGGCAGGGAGAAAAATAGGTTTC	83	NM_001037837.2
TNF-α-R	GCTTTTACTATGGGGTAACCAACTC		
IFN-γ-F	ATGTAGCTGACGGTGGACCT	102	NM_205149.2
IFN-γ-R	CCAAGTACATCGAAACAATCTGGC		
IL-12-F	AAGTAGACTCCAATGGGCAAATG	66	NM_213571.1
IL-12-R	ACGTCTTGCTTGGCTCTTTATAGC		
CXCLi1-F	GGCTGGAGCAAAAGGTATGG	58	NM_205018.2
CXCLi1-R	GCACTGGCATCGGAGTTCA		

## Data Availability

The original contributions presented in this study are included in the article, further inquiries can be directed to the corresponding author.
